# Optimizing COVID-19 vaccine allocation considering the target population

**DOI:** 10.3389/fpubh.2022.1015133

**Published:** 2023-01-06

**Authors:** Zongliang Wen, Tingyu Yue, Wei Chen, Guanhua Jiang, Bin Hu

**Affiliations:** ^1^School of Public Health, Xuzhou Medical University, Xuzhou, China; ^2^Affiliated Hospital of Xuzhou Medical University, Xuzhou, China; ^3^School of Management, Xuzhou Medical University, Xuzhou, China

**Keywords:** COVID-19, vaccine allocation, the target population, multiple-dose vaccine, mixed-integer programming

## Abstract

Vaccine allocation strategy for COVID-19 is an emerging and important issue that affects the efficiency and control of virus spread. In order to improve the fairness and efficiency of vaccine distribution, this paper studies the optimization of vaccine distribution under the condition of limited number of vaccines. We pay attention to the target population before distributing vaccines, including attitude toward the vaccination, priority groups for vaccination, and vaccination priority policy. Furthermore, we consider inventory and budget indexes to maximize the precise scheduling of vaccine resources. A mixed-integer programming model is developed for vaccine distribution considering the target population from the viewpoint of fairness and efficiency. Finally, a case study is provided to verify the model and provide insights for vaccine distribution.

## 1. Introduction

The novel coronavirus pneumonia (COVID-19) began ravaging the globe in 2019 and became a public health emergency. The COVID-19 pandemic threatens global health and economic development. Vaccination is one of the most effective solutions. Rapid development, distribution, and vaccinating the global population may be the most effective way to quell the epidemic (1). The United Nations highlights the importance of providing COVID-19 vaccine as a global public good that is globally accessible and affordable (2). However, the early production capacity of the COVID-19 vaccine has not been able to meet the actual demand, which means that not everyone will be able to get the vaccine immediately in the early stages of use. How to properly distribute COVID-19 vaccines among different geographic regions with limited production capacity has become an urgent scientific issue.

When a major public health emergency occurs, optimization of vaccine allocation is vital to ensuring the safety of people's lives and property (3). COVID-19 vaccination is a large-scale social activity. This nationwide vaccination campaign is unprecedented. However, the distribution of COVID-19 vaccines as special medical supplies is different from ordinary emergency relief supplies such as tents and food. When natural disasters occur, the affected areas, especially those that are more severely affected, need emergency supplies to ensure their safety, and rescue operations cannot be delayed. When the epidemic occurs, not everyone is willing or able to receive the vaccine. Therefore, policy makers need to consider the vaccination willingness and physical fitness of members of the society when allocating vaccines. It is foreseeable that there will remain a significant gap in global production capacity for COVID-19 vaccines over the next 2 to 3 years, and COVID-19 vaccines will remain a scarce resource with limited supply for some time to come (4). In the early stages of the vaccine in use, the number of vaccines may be more nervous, not everyone can be vaccinated. Dividing the priority groups for vaccination and ensuring that those with higher priority levels are the first to receive the vaccine are conducive to the prevention and control of the epidemic. In addition, the government should take some regulatory actions and restrictions to achieve a better epidemic control effect. Therefore, it is essential that policy makers consider prioritization, social policy and other factors. Obviously, the vaccine distribution strategy is very complicated in the selection of target population and the distribution of quantity.

If the society has already entered the vaccination phase when the vaccine is distributed, then the target population includes unvaccinated and vaccinated individuals who have not completed the whole vaccination process. Vaccination adheres to the principle of “informed, consensual and voluntary”, and requires the active cooperation of social members to establish a crowd immune barrier. Therefore, we first consider the target population when allocating vaccines and analyze them from multiple perspectives. In the context of limited vaccination capacity, optimizing vaccine allocation and prioritization is critical to achieving herd immunity and restoring normal living standards before the pandemic (5). We are also considering the impact of the COVID-19 pandemic on the mental health of the population (6). With the aim to ensure the fairness and efficiency of vaccination, this paper studies the allocation of vaccines considering characteristics of the vaccinated population, attitude toward the vaccine, priority groups for vaccination, and vaccination priority policy simultaneously. We propose a mixed-integer linear programming model of vaccine distribution considering vaccinated populations to maximize vaccination coverage by combining actual vaccine supply with vaccination policies.

Summarily, the main contributions of this paper can be denoted as follows:

**Contribution 1:** From the perspective of the vaccinated population, we consider the vaccination willingness, the priority of the target population and the relevant policies of the society on the vaccination campaign when allocating vaccines. We consider that some people cannot or do not want to be vaccinated, divide the vaccinated population into different priority levels, and give priority to people who have received *n* dose(s) of vaccine following relevant vaccination policies.

**Contribution 2:** From the perspective of quantity allocation, we use multiple doses of COVID-19 vaccines as the allocated resource, and combine with realistic conditions such as inventory and cost. It is more general and applicable to depict multi-dose vaccine allocation into the optimization model.

**Contribution 3:** From the perspective of fairness and efficiency of COVID-19 vaccine distribution, this paper considers the particularity of vaccination groups and the efficiency of quantitative allocation of vaccines. We develop a vaccine distribution optimization model under the two-level network of “point-of-supply – point-of-demand”. The study follows the forefront, conforms to the reality, and improves the vaccine resources allocation system.

The remaining of the paper is organized as the follows. Section 2 reviews the literature related to the target population, vaccine quantity allocation, and epidemic resource distribution. The problem description and model hypothesis are presented in Section 3. Section 4 develops a vaccine scheduling optimization model under the COVID-19 pandemic. Section 5 uses a real case to verify the model and conduct sensitivity analysis by adjusting the budget. Section 6 discusses the implications of management and the generality of the model. Finally, Section 7 summarizes the research of this study.

## 2. Literature review

This study is related to three streams of literature, namely, the target population, allocation of vaccines, and resource allocation under the COVID-19 pandemic.

The literature on the attitude and priorities of the target vaccination population is related. Under an epidemic situation, vaccination is an effective plan to prevent infectious diseases. Even in a very severe situation, people still have vaccine hesitancy. Vaccine hesitancy refers to a lack of public trust in vaccination, culminating in delays or refusals to vaccinations, and even boycotts to undermine vaccination efforts. In Mahmud's survey (7), 61.16% of respondents were willing to receive the COVID-19 vaccine. Only 35.14% said they were willing to receive the COVID-19 vaccine immediately in the receiving group. However, 64.86% were likely to delay vaccination until they could determine the effectiveness and safety of the vaccine. Additionally, in a survey in France, Guillon et al. (8) found that only 30.5% of respondents agreed to be vaccinated against COVID-19 in the first phase of 2021, whereas 31.1% expressed uncertainty. Goel's survey (9) shows that more than 60% of respondents in India have negative attitudes toward COVID-19 vaccines. Vaccine hesitancy exists in all countries and regions, which affects vaccination rates.

Developing and using vaccines for COVID-19 are of great concern to all countries, and different regions affected by the epidemic may require different approaches to prioritize vaccination. In the early stage of vaccine use, the number of vaccines may be relatively tight, not everyone can be vaccinated. Different scholars may use different methods to determine the priority of vaccination population. Hogan et al. (10) found that the impact of SARS-CoV-2 vaccination on health depends on different factors. The best distribution strategy for limited vaccines within a country is to vaccinate the elderly. In contrast, in the case of a large supply, distribution can be shifted to key spenders to protect vulnerable groups indirectly. Emanuel et al. (11) mentioned in their study that equitable distribution of vaccines is a controversial issue of distribution; thus, proposing three fundamental values of vaccine distribution, which are benefiting people and limiting harm, prioritizing vulnerable groups, and equal moral care. Weiss et al. (12) believe that people with moderate vulnerability need to be given higher priority and potentially exposed populations given greater weight. Sabatino et al. (13) suggested that vaccine distribution policies should prioritize individuals at the highest risk of adverse outcomes for COVID-19, provide overall evidence of exposure and clinical risk of patients with adult congenital heart disease (ACHD), and propose to incorporate risk profiles of these patients into vaccine distribution decisions. Furthermore, Wilbrink (14) compared the implementation of different vaccine distribution strategies to analyze which priority strategies introduced would most effectively recover these populations and control COVID-19 outbreaks. Results showed that prioritizing the elderly or high-risk transmission groups is effective. Foy et al. (15) used an age-structured extended SEIR model with a social connection matrix to evaluate age-specific vaccine distribution strategies in India. They found that prioritizing COVID-19 vaccine allocation to older populations led to the greatest relative reduction in the number of deaths. Paloyo et al. (16) pointed out that for the Philippines and some other countries with limited supply, the elderly may not always be prioritized. Moradi et al. (17) used an online survey to assess Iranian population's views on priority individuals and groups for COVID-19 vaccination. They found that healthcare workers, high-risk patients, and the elderly were prioritized vaccination groups. There is a growing consensus that sickest or medically most vulnerable people should be prioritized for vaccination. Studies have shown that priority is based on the vulnerability of social groups, such as the homeless or “hard-to-reach” groups. Vulnerable groups are prone to some difficulties and obstacles in obtaining vaccines, but in fact their living conditions are more in need of vaccine protection. Despite initial limited vaccine supplies, the CDC's phased vaccination guidelines help protect those most vulnerable to COVID-19 (18). Notably, vaccine hesitancy is more pronounced among vulnerable populations. We found that scholars divided priority populations from different perspectives or according to different criteria, resulting in different classification results. In general, when the number of vaccines is limited, the priority group strategy has certain advantages compared with the random vaccination strategy of the whole population.

Our study is closely related to the literature on the allocation of vaccines. Transportation of vaccines can guarantee the safety of the affected people for the first time, which is related to recognizing the national policy by the people of all countries. Some researchers have mainly used epidemiological models to predict the spread of viruses at national and regional levels (19). For example, Yu et al. (20) proposed a new SEIR model, called the hybrid SEIR-V model, which considers the infection status of host populations in different age groups and describes the dynamic characteristics of virus transmission in different geographical locations. However, Enayati and Özaltin (21) focused on the optimal distribution of influenza vaccines in heterogeneous populations and adopted the influenza transmission model to effectively extinguish emerging outbreaks at an early stage. Liu et al. (22) used the SEIR model to describe the dynamic epidemic diffusion process. The goal is to minimize the total logistics cost of healthcare resource allocation, and a heuristic algorithm is designed to solve the proposed model. Lee et al. (23) proposed a new multi-population mean-field control model and explained how population movement and vaccine distribution are integrated into the constraint optimization problem. Additionally, Yin and Büyüktahtakin (24) proposed a data-driven, multi-stage, stochastic epidemic-vaccine-logistics model that can assess the growth scenario of each disease under risk metrics to optimize the distribution of treatment centers, resources, and vaccines. It is also possible to minimize the number of common infections, deaths, and close contacts of infected persons within a limited budget. Rastegar et al. (25) proposed a mixed-integer linear programming model for equitable distribution of influenza vaccine inventory locations in developing countries during a pandemic. The model divides the vaccinated population into different groups, distributing vaccines to key healthcare providers, the elderly, pregnant women, and others. In recent years, studies on vaccine allocation have been active in the epidemic context. However, due to the rapid spread of COVID-19 and limited vaccine resources, COVID-19's vaccine allocation system still needs to be further improved.

The joint response of governments, healthcare providers, and the public to COVID-19 is now a top priority. Also, the outbreak has led to extensive research by experts and academics globally on vaccine resource allocation during the pandemic. Grauer et al. (26) proposed a strategy for vaccine distribution, which sequentially prioritizes the areas with the highest number of new infections within a given time frame and compares this scheme with the standard practice of distributing vaccines by population. Fu et al. (27) proposed an epidemiological model, which expressed the robust epidemiological optimization model of risk index minimization as a mixed-integer linear optimization problem through appropriate approximation. They applied the robust epidemiological optimization model to allocate vaccines within a given vaccination budget. Katherine Klise and Bynum (28) developed a facility location optimization model to integrate some key information to help decision-makers determine the best site to build a facility to meet expected resource needs. Tavana et al. (29) proposed a mixed-integer linear programming model for equitable distribution of COVID-19 vaccines in developing countries. Furthermore, they divided vaccines into cold, extremely cold, and ultra-cold categories, with specific refrigeration required to store and distribute different vaccines. Buhat et al. (30) used a non-linear model (NLP) to determine the optimal allocation of COVID-19 test kits in the Philippines to provide a fair opportunity for all infected persons to be tested. Medlock and Galvani (31) identified the optimal vaccine allocation using a model based on survey data and parameterized influenza pandemic mortality data.

There are arising studies that focus on the allocation of vaccine resources. However, compared with research on emergency medical supplies scheduling, in the context of large-scale epidemics, studies on the field of vaccine allocation optimization are relatively few. None of the studies concern the situation of the target population for vaccination, and directly distribute vaccines to the lower level as ordinary emergency supplies, which is easy to cause unfair distribution and waste of resources. Therefore, this paper considers the priority of the vaccination population, vaccination attitude and related policies. It combines the fairness and efficiency of COVID-19 vaccine distribution to explore the precise scheduling strategy of allocating vaccines from the concentration center to the target population when the number of vaccines is limited.

## 3. Problem description and model assumptions

### 3.1. Problem description

When COVID-19 vaccines become available, governments should prepare for new challenges (32). The scarcity of vaccine resources requires effective resource allocation strategies.

(1) Selection of priority groups for the target population: Equitable allocation does not always mean the average distribution among individuals, but rather a distribution in which “people who need more enjoy higher priority than those who need less.” In some foreign countries, vaccination groups are divided according to age, and the elderly prioritize vaccination. For example, in Norway, the elderly are prioritized for protection. The attending doctor evaluates the elderly and infirm before deciding whether to be vaccinated. In China, the “high-risk” elderly population was not prioritized in the early stage of vaccine use. Those aged between 18 and 59 who need emergency vaccination will be prioritized. In practice, priority is given to target groups depending on containment and vaccine availability. However, in general, in view of the shortage of vaccine resources in the early stage of the outbreak, it is necessary to give priority to some population groups in the target population for vaccination, and the division of priority vaccination population groups is one of the considerations of the model in this study.
(2) Attitude toward the target population: In reality, some people with contraindications to COVID-19 vaccines are unsuitable for vaccination. There are also some people who are hesitant or even refuse vaccination due to the influence of vaccine factors, personal factors and cognitive factors. Therefore, in the process of vaccine distribution, people who cannot or will not be vaccinated are excluded and the vaccine is distributed to those who actually need it.
(3) Priority is given to those who have received *n* dose(s) of the vaccine: If the distribution of multiple doses of vaccine has been in the vaccination phase, then the society includes the vaccinated population (*n* = *N*) and the population to be vaccinated (0 ≤ *n*<*N*). The target population of vaccination mentioned in this paper mainly refers to the population to be vaccinated with negative antibody, that is, the population who has not been vaccinated (*n* = 0) and the population who has been vaccinated but has not completed the whole course of vaccination (0 < *n*<*N*). For *N* doses of vaccine, people who receive *n* dose(s) of vaccine need to receive (*N*- *n*) dose(s) of vaccine to complete the full course of vaccination.

According to the needs of COVID-19 prevention and control, different regions have different priority vaccination policies. Take China as an example, for a period of time, the first dose of COVID-19 inactivated vaccine has been suspended in many regions of the country, and the second dose of the vaccine is guaranteed. That is, the vaccine is given priority to the population who has received one dose (*n* = 1), so as to ensure the full vaccination of the vaccine and establish the immune barrier of the population. In Canada, in addition to ensuring a second dose is given to high-risk groups, such as health care workers, the first dose of vaccines is encouraged to the general population. In Canada and some other regions, in order to ensure that more people in the unvaccinated population receive at least one dose of COVID-19 vaccine, in addition to ensuring that high-risk groups, such as health care workers, receive the second dose, the vaccine is encouraged to be prioritized to the unvaccinated population (*n* = 0). There are no universal answers to the intense debate about how best to use existing vaccines (33). Therefore, the model parameters can be set according to the relevant regional policies to ensure the priority of vaccine allocation to the population vaccinated with n injection, so that the vaccine can be more reasonably distributed in the case of limited resources, so as to achieve better immunization effect.

**Figure 1:**
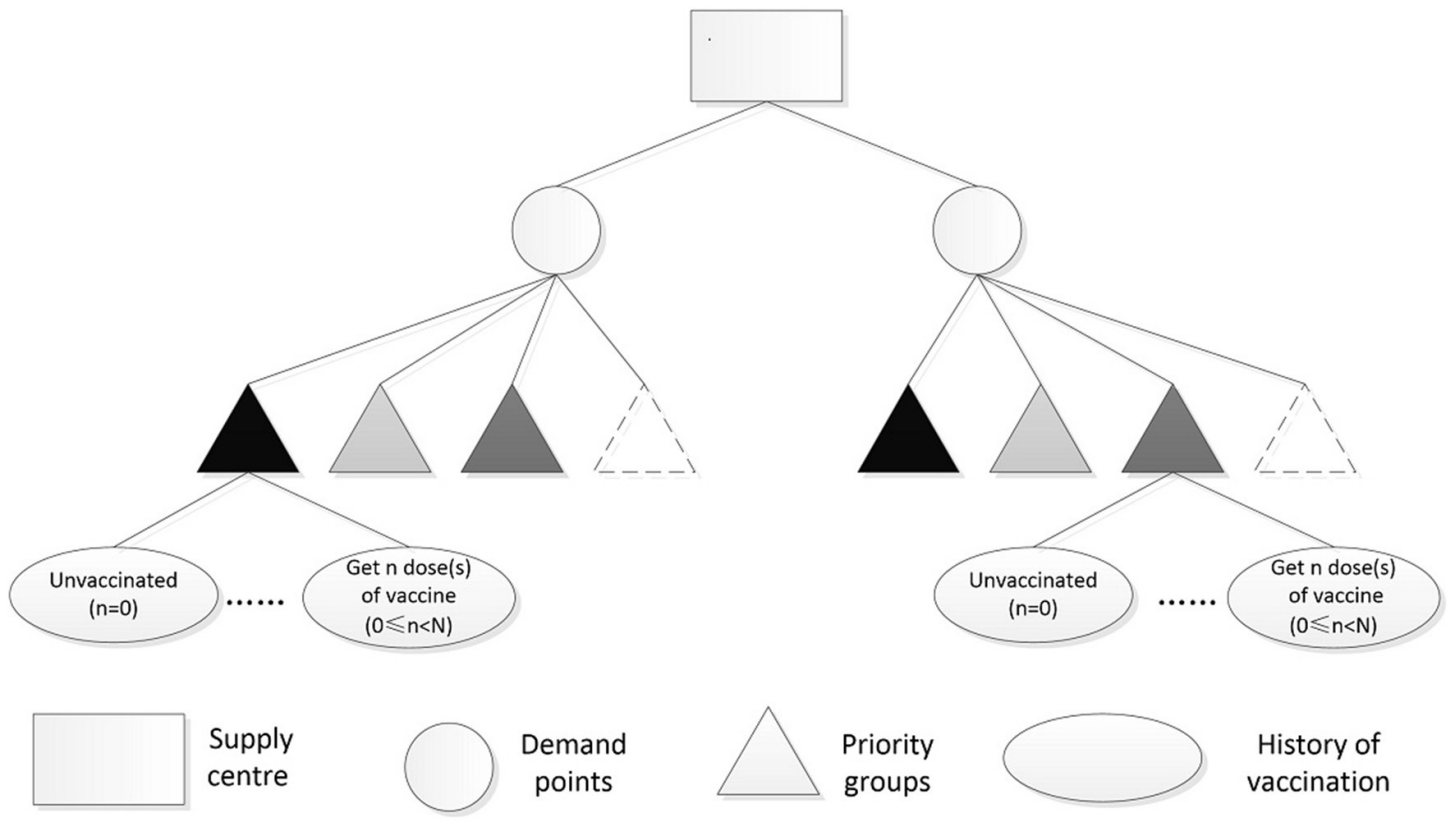
Distribution of COVID-19 vaccine.

Under the background of limited number of vaccines in the early stage of use and herd immunity in all regions, this study develops a vaccine distribution model considering the target population based on the fairness and efficiency of COVID-19 vaccine distribution and the goal of maximizing vaccine coverage. Vaccines are distributed and scheduled under a two-level “supply-demand-point” network, such as “provincial centralized distribution center - municipal Centers for Disease Control (CDC)” “municipal centralized distribution Center - District and county CDC,” and other networks. According to the quantity of vaccines purchased and supplied, decision makers can accurately and scientifically allocate vaccines and timely distribute them to all vaccination sites to fully guarantee the implementation of vaccination work. Figure 1 shows the diagram of COVID-19 vaccine distribution studied in this paper. The icon with darker colors indicates a higher priority for each priority group, and the icon with a dotted line represents people who are unable or unwilling to be vaccinated.

The mathematical model mainly considers:

- Vaccination history of the target population.
- Different levels of priority groups for vaccination.
- Some people are unable or unwilling to be vaccinated.
- Priority should be given to the population vaccinated with n dose(s) of vaccine.

### 3.2. Model assumptions

(1) The type of COVID-19 vaccine allocated is a multi-dose vaccine.
(2) People at all levels can be vaccinated simultaneously, and there is no such thing as vaccinating only one group.
(3) If there is a repetition between groups of the target population, they are divided into the higher priority group.
(4) The demand for vaccines is fixed for a period of time.
(5) Demand points are independent of each other, vehicles are delivered in one direction, and there is no material transfer.
(6) The number of vehicles transporting materials is sufficient, and the road condition is good. The road speed, approved load and transport cost per unit distance of the vehicles are the same.

## 4. Mathematical model

### 4.1. Symbol description

Table 1 shows the list of mathematical notations, which mainly includes the sets, parameters and variables of the model.

**Table 1:** List of mathematical notations.

**Sets**	
*I*	Set of demand points
*J*	Set of priority groups
*N*	The target total number of doses to be completed for the entire course of vaccination of a type of vaccine
*K*	Set of costs
**Parameters**	
*i*	Demand points, *i* ∈*I*
*j*	Priority groups, *j* ∈*J*
*n*	The dose of vaccine that has been administered to the target population (0 ≤ *n*<*N*)
*k*	Types of costs for vaccine distribution
*P*	Total population of all regions
*p_*ij*_*	The number of the target population in group *j* in demand point *i*
*p_*ijn*_*	The number of the target population who had received *n* dose(s) of the vaccine in group *j* in demand point *i*
*μ_*ijn*_*	The proportion of the target population with *n* dose(s) who will not receive the vaccine in group *j* in demand point *i*
*α_*n*_*	The expected proportion of people who have been vaccinated with *n* dose(s)
*θ_*j*_*	Minimum vaccination coverage in group *j*
*Q*	The total amount of vaccines in the supply center
*Q_*i*_*	Capacity of vaccine storage in warehouse of demand point *i*
*TC_*ik*_*	Type *k* cost of a single dose of vaccine in demand point *i*
*BG*	Budget
**Variables**	
*y_*ijn*_*	Integer, the number of individuals vaccinated in group *j* vaccinated with *n* dose(s) in area *i*
*x_*ijn*_*	Integer, the number of vaccines allocated in group *j* in the area *i* vaccinated with *n* dose(s)

### 4.2. Optimization model for vaccine allocation for COVID-19


(1) $$\begin{array}{l} Max\;Obj\text{=}\underset{i\in I}{\Sigma }\underset{j\in J}{\Sigma }\underset{n\in N}{\Sigma }\frac{y_{ijn}}{p} \end{array}$$

s.t.


(2) $$\begin{array}{l} y_{ijn}\leq p_{ijn}\text{(1-}\mu_{ijn}\text{)},\forall i\in I,j\in J,n\in N \end{array}$$

(3) $$\begin{array}{l} \frac{\underset{n\in N}{\Sigma }y_{ijn}}{p_{ij}}\geq \theta_{j},\forall i\in I,j\in J \end{array}$$

(4) $$\begin{array}{l} y_{ijn}\geq p_{ijn}\text{(1-}\mu_{ijn}\text{)}\alpha_{jn},\forall i\in I,j\in J \end{array}$$

(5) $$\begin{array}{l} x_{ijn}=\left(N-n\right)\;{\text{y}}_{ijn}\text{,}\forall i\in I,j\in J,n\in N \end{array}$$

(6) $$\begin{array}{l} \underset{i\in I}{\Sigma }\underset{j\in J}{\Sigma }\underset{n\in N}{\Sigma }x_{ijn}\leq Q\text{,}\forall i\in I,j\in J,n\in N \end{array}$$

(7) $$\begin{array}{l} \underset{j\in J}{\Sigma }\underset{n\in N}{\Sigma }x_{ijn}\leq Q_{i}\text{,}\forall i\in I \end{array}$$

(8) $$\begin{array}{l} \underset{i\in I}{\Sigma }\underset{j\in J}{\Sigma }\underset{k\in K}{\Sigma }\underset{n\in N}{\Sigma }TC_{ik}x_{ijn}\leq BG \end{array}$$

(9) $$\begin{array}{l} y_{ijn},x_{ijn}\in Z^{+},\forall i\in I,j\in J,n\in N,k\in K \end{array}$$

Objective function (1) maximizes the vaccination rate of COVID-19 vaccines and makes vaccination available to many people to achieve herd immunity. Constraint (2) ensures that the number of vaccinated individuals in group *j* in the area *i* vaccinated with *n* dose(s) should not exceed the number of target population in group *j* in the area *i* vaccinated with *n* injection. The actual target population is the population to be vaccinated except those who cannot or will not be vaccinated. Constraint (3) ensures that the vaccination ratio for each priority in each region is not lower than the minimum pre-set coverage rate for each level to prevent low vaccination rates at some demand points. Constraint (4) indicates that people in group *j* who have received *n* (0 ≤ *n*<*N*) injection(s) are given priority. Constraint (5) represents the relationship between the amount of vaccine allocated and the number of people vaccinated, considering vaccination history. Constraints (6) and (7) are inventory and capacity constraints, respectively. Constraint (6) is the constraint that the total allocation of vaccines in all demand points does not exceed the vaccine stock in the allocation center. Additionally, Constraint (7) is that the amounts of vaccines obtained in each region does not exceed its warehouse capacity. Constraint (8) indicates that the total cost of vaccine distribution and vaccination in each region does not exceed the local budget. The total cost, such as transportation, storage, and personnel, should be included. Constraint (9) means the decision variables are a positive integer.

## 5. Case study

### 5.1. Case background

The COVID-19 epidemic caused a public health crisis and had a significant social, political, and economic impact worldwide. The pace of development of many COVID-19 vaccine candidates is rapid that countries are beginning to approve them for mass distribution. However, there may be some difficulties, such as the insufficient supply of COVID-19 vaccines. In one distribution, the Xuzhou municipal government received 10 million doses of inactivated COVID-19 vaccine and distributed them to ten regions under its jurisdiction. Due to the limited number of vaccines, it is necessary for policy makers to formulate reasonable distribution strategies with both fairness and efficiency in mind. It's worth noting that some of the model parameters are based on reasonable estimates as detailed vaccine cost and other relevant data are not published officially, and these values parameters do not affect the nature of the model.

When vaccine availability is limited, resources should be more skewed toward a subset of the population. In Table 2, According to the standards published by China's National Health Commission, the target population in China is roughly divided into three groups: high-risk population, high-danger population, and the general population. This classification scheme based on risk and impact of infection can avoid the problems of inconsistency where the division of priority vaccination population among different regions is inconsistent due to outbreak control and vaccine supply situations. High-risk groups mainly refer to epidemic prevention and medical personnel, entry-exit workers, people who need to go to high-risk countries for special reasons, and people who play an important role in fighting the epidemic. High-danger groups, mainly the elderly, young children, pregnant women, and people with a low resistance to COVID-19, have a high-risk of infection.

**Table 2:** Number of priority persons in each region.

**Priority level**			
	**High-risk group**	**High-danger group**	**General population**
Gulou district	126,717	190,076	316,793
Yunlong district	75,596	113,394	188,990
Jiawang district	103,571	155,357	258,928
Quanshan district	114,316	171,474	285,791
Tongshan district	266,632	399,948	666,581
Pizhou city	387,514	581,271	968,786
Xinyi city	225,858	338,786	564,644
Suining county	288,317	432,476	720,793
Feng county	241,945	362,918	604,864
Pei county	248,216	372,324	620,540

The priority vaccination population was divided into three groups, and the priority order was as follows: high-risk population > high-danger population > the general population. The higher the priority, the greater the risk of infection and the more severely affected by COVID-19. They are more in need of vaccination than the general population and should therefore be given priority when vaccine availability is limited. However, if the general population cannot be vaccinated, they may experience panic and jealousy. Therefore, the decision makers also need to allocate a certain amount of vaccine to the general population when making vaccine allocation. We set the corresponding minimum vaccine coverage for each group, and the group with higher priority had higher minimum coverage than the group with lower priority, as shown in Table 3.

**Table 3:** Minimum coverage for each priority.

	**High-risk group**	**High-danger group**	**General population**
Minimum coverage	0.95	0.7	0.5

Table 4 shows the inoculation status of each region. When vaccine availability is limited, priority is promoted for those who have received one dose of vaccine. For vaccinations have been vaccinated but not completed group and unvaccinated people, the vaccine coverage rates are 3/5 and 2/5 respectively. The purpose is to give priority to vaccinated personnel who have received one shot of vaccine, so as to achieve better immune effect. At the same time, in order to avoid panic and jealousy among unvaccinated people, a certain amount of vaccine will also be appropriately allocated to such people.

**Table 4:** Vaccination in each region.

**High-risk group**			**High-danger group**		**General population**	
	**Get a dose**	**Unvaccinated**	**Get a dose**	**Unvaccinated**	**Get a dose**	**Unvaccinated**
Gulou district	76,030	50,687	114,045	76,030	190,076	126,717
Yunlong district	45,358	30,238	68,036	45,358	113,394	75,596
Jiawang district	62,143	41,428	93,214	62,143	155,357	103,571
Quanshan district	68,590	45,726	102,885	68,590	171,474	114,316
Tongshan district	159,979	106,653	239,969	159,979	399,948	266,632
Pizhou city	232,509	155,006	348,763	232,509	581,271	387,514
Xinyi city	135,515	90,343	203,272	135,515	338,786	225,858
Suining county	172,990	115,327	259,485	172,990	432,476	288,317
Feng county	145,167	96,778	217,751	145,167	362,918	241,945
Pei county	148,929	99,286	223,394	148,929	372,324	248,216

Vaccine distribution also faces vaccine hesitancy. Vaccine hesitancy is universal in all countries and COVID-19 vaccine hesitancy has been a growing concern (34). The decision makers need to consider the vaccination intention of the target population when making allocation decisions. According to the existing literature on vaccination willingness and society's current situation, the proportion of high-risk, high-danger, and the general population who are unable or unwilling to be vaccinated is set as 0.016, 0.034, and 0.020, respectively. Therefore, by calculation, the number of people to be vaccinated in each region is shown in Table 5.

**Table 5:** Actual number of people to be vaccinated in each region.

**High-risk group**			**High-danger group**		**General population**	
	**Get a dose**	**Unvaccinated**	**Get a dose**	**Unvaccinated**	**Get a dose**	**Unvaccinated**
Gulou district	76,030	49,876	114,045	73,445	190,076	124,183
Yunlong district	45,358	29,755	68,036	43,815	113,394	74,084
Jiawang district	62,143	40,766	93,214	60,030	155,357	101,500
Quanshan district	68,590	44,995	102,885	66,258	171,474	112,030
Tongshan district	159,979	104,946	239,969	154,540	399,948	261,300
Pizhou city	232,509	152,526	348,763	224,603	581,271	379,764
Xinyi city	135,515	88,898	203,272	130,907	338,786	221,340
Suining county	172,990	113,482	259,485	167,109	432,476	282,551
Feng county	145,167	95,230	217,751	140,232	362,918	237,106
Pei county	148,929	97,698	223,394	143,866	372,324	243,251

Tables 6, 7 show the warehouse capacity and the cost of vaccine distribution in each region respectively. Among them, the cost types mainly include transportation cost, storage cost, and personnel cost in this case. The total budget for vaccination services and distribution is 150,000,000 yuan.

**Table 6:** Storage capacity of each region.

	**Gulou district**	**Yunlong district**	**Jiawang district**	**Quanshan district**	**Tongshan district**
Storage capacity	1,260,000	900,000	1,000,000	1,000,000	2,600,000
	Pizhou city	Xinyi city	Suining county	Feng county	Pei county
	7,600,000	5,000,000	6,000,000	5,000,000	6,000,000

**Table 7:** Transportation cost, storage cost, and personnel cost in each region.

	**Transportation cost**	**Storage cost**	**Personnel cost**
Gulou district	2.2	5	4
Yunlong district	2.3	4.5	4
Jiawang district	2.8	3.5	4
Quanshan district	2.9	3.5	4
Tongshan district	3	7.5	4
Pizhou city	6	10	3
Xinyi city	7.5	6.5	3
Suining county	7.2	7.5	3
Feng county	6.5	7	3
Pei county	6.7	7	3

### 5.2. Result analysis

MATLAB and LINGO were used to calculate the results of the model. The results are shown in Tables 8, 9.

**Table 8:** Number of vaccinated individuals in each region.

	**High-risk group**		**High-danger group**		**General population**	
	**Get a dose**	**Unvaccinated**	**Get a dose**	**Unvaccinated**	**Get a dose**	**Unvaccinated**
Gulou district	76,030	49,876	114,045	73,445	190,076	124,183
Yunlong district	45,358	29,755	68,036	43,815	113,394	74,084
Jiawang district	62,143	40,766	93,214	60,030	155,357	40,600
Quanshan district	68,590	44,995	102,885	66,258	171,474	44,812
Tongshan district	159,979	93,322	239,969	61,816	239,970	104,520
Pizhou city	232,509	135,629	317,049	89,841	348,763	151,906
Xinyi city	135,515	79,050	184,787	52,363	203,272	88,536
Suining county	172,990	100,911	235,890	66,843	259,485	113,020
Feng county	145,167	84,681	217,751	56,093	217,751	94,843
Pei county	148,929	86,876	216,775	57,546	223,394	97,301

**Table 9:** Distribution of vaccines by region.

	**High-risk group**		**High-danger group**		**General population**	
	**Get a dose**	**Unvaccinated**	**Get a dose**	**Unvaccinated**	**Get a dose**	**Unvaccinated**
Gulou district	76,030	99,752	114,045	146,890	190,076	248,366
Yunlong district	45,358	59,510	68,036	87,630	113,394	148,168
Jiawang district	62,143	81,532	93,214	120,060	155,357	81,200
Quanshan district	68,590	89,990	102,885	132,516	171,474	89,624
Tongshan district	159,979	186,644	239,969	123,632	239,970	209,040
Pizhou city	232,509	271,258	317,049	179,682	348,763	303,812
Xinyi city	135,515	158,100	184,787	104,726	203,272	177,072
Suining county	172,990	201,822	235,890	133,686	259,485	226,040
Feng county	145,167	169,362	217,751	112,186	217,751	189,686
Pei county	148,929	173,752	216,775	115,092	223,394	194,602

The optimal result is 71.86%, meaning that when we consider the constraint conditions of vaccine storage, budget, and minimum coverage, the maximum vaccination rate is 71.86%, with 7,468,263 vaccinated people and 9,775,979 doses of vaccine allocated. As can be seen from Table 8, the allocation of vaccines in each priority group almost covers the high-risk group, and is distributed to the high-risk group and the general population in each region according to the proportion of priority. This result validates the validity of the model. As we would expect to be able to distribute the vaccine to the vast majority of the high-risk population, the majority of the high-risk population, and a certain number of the general population with limited amounts of vaccine. In line with the principle of voluntary vaccination, the majority of the vaccine will be given as a priority to those who have received a single dose and a smaller proportion will be given to those who have not yet received the vaccine, in accordance with local social policies. Table 9 shows the number of vaccines allocated for each region.

### 5.3. Sensitivity analysis

Five scenarios were considered in the sensitivity analysis, including budget increase and decrease. The corresponding results of these five scenarios are shown in Table 10. As the budget increases, the number of vaccines to be allocated and vaccinated individuals increases. Thus, increasing vaccine coverage makes it easier to achieve herd immunity through increased budgets; conversely, as budgets decrease, the number of vaccines available will decrease, corresponding to a decrease in the number of people vaccinated and a decrease in vaccine coverage. Therefore, policy makers need to make trade-offs between cost and vaccination coverage when allocating vaccines. When the epidemic is severe and the demand for vaccines is high, governments need to increase funding to increase vaccine coverage and establish a universal immunization barrier as much as possible. For low-risk areas with a low budget and a small number of vaccines, the government can temporarily vaccinate high-risk groups and high-risk groups, and then gradually move to the general population.

**Table 10:** Sensitivity analysis of budget changes.

**Scenario**	**Budget**	**Coverage**	**The number of vaccination**	**Number of vaccines allocated**
S1	145,000,000	68.93	7,164,318	9,357,274
S2	147,500,000	70.63	7,340,562	9,585,076
S3	150,000,000	71.86	7,468,263	9,775,979
S4	152,500,000	72.67	7,552,669	9,888,246
S5	155,000,000	74.25	7,716,854	10,000,000

## 6. Discussion

The vaccine distribution model constructed in this study mainly includes the following parts: (i) multi-dose vaccine distribution. For the multi-dose COVID-19 vaccine type, there are unvaccinated individuals and those who have not completed the full vaccination in the society during a certain period, so the vaccination history of the vaccinated individuals should be considered when allocating the vaccine. (ii) The vaccination population can be divided into different priority levels to distribute more vaccines to the higher priority groups. (iii) Additionally, there are still individuals who are unable or unwilling to be vaccinated. Following the policy and principle of voluntary vaccination, vaccine distribution should exclude such groups to avoid wasting resources and allocate vaccine resources to those who are able and willing to be vaccinated. (iv) In accordance with regional policies, priority should be given to the population vaccinated with *n* dose(s) of vaccine. (v) A vaccine distribution model was developed to account for the vaccinated population under COVID-19 considering equity and efficiency. This paper takes into account fairness and efficiency, and establishes a vaccine distribution model considering the target population of vaccination under COVID-19, which has certain practical significance. First, the goal of the model is to maximize the vaccination rate. Herd immunity is easier to achieve when the number of people vaccinated is larger. Some people with contraindications or who are not in the age range for vaccination are not suitable for vaccination, which requires that those who are suitable for vaccination should be covered. At the same time, the willingness to vaccinate is one of the factors affecting the vaccination rate. The government should take such measures as opening lectures and publicity to enhance the public's vaccination awareness, so as to improve the vaccination rate. In addition, the division of vaccine priority groups, the implementation of vaccination-related policies, and the appropriate “skewering” of vaccine allocation to groups with greater need can ensure the fairness of distribution. High-risk groups are the backbone of the epidemic prevention work and the maintenance of social order. High-danger groups are vulnerable groups that need to be paid close attention to, and their protection is of great significance. This paper also considers the efficiency of general material distribution, such as warehouse capacity, material reserves, cost, etc. should be completed under the constraints of total inventory, total budget and other conditions. The optimal model of vaccine distribution established in this paper can provide a flexible vaccine distribution scheme and provide a reference for decision makers to formulate effective vaccine distribution strategies.

According to the technical guidelines for new coronavirus vaccination, vaccine types mainly include adenovirus vector vaccine, inactivated vaccine and recombinant protein vaccine. One injection (*N* = 1), two injections (*N* = 2) and three injections (*N* = 3) are required to complete the whole vaccination process. The distribution of single dose vaccine is relatively simple, while the distribution of multi-dose vaccine in this study not only takes into account the willingness to vaccinate and priority vaccination population, but also involves considering vaccination history and prioritizing the distribution of vaccine to the population that has been vaccinated with *n* dose(s) according to relevant policies. While the allocation optimization model of multi-dose vaccine can also be applied to the allocation of single-dose vaccine, in which case, constraints (4) and (5) are unnecessary and can be omitted. Because when the allocated resources are single-dose vaccines (*N* = 1), there is only one group of target vaccinated people, that is, the unvaccinated population (*n* = 0). In this case, there is no situation in the society that has been vaccinated but has not completed the whole process of vaccination, which means that there is no need to give priority to those who have received n-dose of vaccine, and there is no situation that people who have been vaccinated but have not completed the whole process of vaccination still need to be vaccinated (*N*- *n*) dose(s).

## 7. Conclusions

In this study, based on the current situation of the epidemic and from the perspective of vaccination history, priority vaccination groups, and the voluntary nature of vaccination, a vaccine distribution model considering vaccination groups was constructed, which was realistic and appropriate, and combined with the different characteristics of vaccination groups, providing decision-makers with a more flexible decision-making tool. The results showed that our model could exclude some people who are unable or unwilling to refuse vaccination and prioritize those with higher priority and those who have been vaccinated but have not completed the full vaccination. Furthermore, the integer programming model was validated with real world data. Through numerical experiments, we also found that increasing the budget can improve the vaccination rate, enable more people to get vaccinated, and achieve herd immunity with a given number of vaccines.

The optimization of vaccine allocation studied in this paper pays more attention to the target population of vaccination. However, vaccine allocation and vaccination process need to be completed over a long period of time, which is a dynamic and multi-cycle distribution process. The static model established in this paper has certain limitations. To further improve the applicability and generality of the model, the future research directions of this paper are as follows: (i) Constructing a multi-cycle vaccine distribution model. The single-cycle model has some limitations in the case of limited vaccine, and the next phase of vaccine allocation according to the previous phase of vaccine use and vaccination is the focus of the following research. (ii) Based on the infectivity of the epidemic, the vaccine distribution model under the situation of uncertain demand was developed to dynamically analyze the vaccine demand to conduct precise scheduling of vaccines more accurately. (iii) Presently, vaccine distribution in many countries is still unfair. Therefore, a model for national and even international large-scale vaccine distribution could be developed in the future.

## Data availability statement

The original contributions presented in the study are included in the article/supplementary material, further inquiries can be directed to the corresponding author.

## Author contributions

Conceptualization and supervision: BH and ZW. Methodology and visualization: TY. Investigation: WC and GJ. Writing—original draft preparation: BH and TY. Writing—review and editing: ZW, TY, WC, GJ, and BH. All authors have read and agreed to the published version of the manuscript.
